# Venetoclax induces deep hematologic remissions in t(11;14) relapsed/refractory AL amyloidosis

**DOI:** 10.1038/s41408-020-00397-w

**Published:** 2021-01-11

**Authors:** Vikram J. Premkumar, Suzanne Lentzsch, Samuel Pan, Divaya Bhutani, Joshua Richter, Sundar Jagannath, Michaela Liedtke, Arnaud Jaccard, Ashutosh D. Wechalekar, Raymond Comenzo, Vaishali Sanchorawala, Bruno Royer, Michael Rosenzweig, Jason Valent, Stefan Schönland, Rafael Fonseca, Sandy Wong, Prashant Kapoor

**Affiliations:** 1 Division of Hematology/Oncology, Columbia University Irving Medical Center, New York, NY USA; 2 The Patricia Lynch Cancer Center, Holy Name Medical Center, Teaneck, NJ USA; 3 Columbia University Irving Medical Center, New York, NY USA; 4 Division of Hematology and Medical Oncology, Tisch Cancer Institute, Mount Sinai Medical Center, New York, NY USA; 5 Stanford Amyloid Center, Stanford University School of Medicine, Stanford, CA USA; 6 Hematology Department, French Reference Center for AL Amyloidosis (Limoges-Poitiers), CHU Limoges, Limoges, France; 7 National Amyloidosis Centre, University College London, London, UK; 8 John C. Davis Myeloma and Amyloid Program, Tufts Medical Center, Boston, MA USA; 9 Amyloidosis Center, Boston University School of Medicine, Boston Medical Center, Boston, MA USA; 10 Paris 7 University and APHP, Immuno-Hematology Department, Saint-Louis Hospital, Paris, France; 11 Department of Hematology and Hematopoietic Cell Transplantation, Judy and Bernard Briskin Center for Multiple Myeloma Research, City of Hope, Duarte, CA USA; 12 Department of Hematology and Medical Oncology, Taussig Cancer Center, Cleveland Clinic, Cleveland, OH USA; 13 Amyloidosis Center, Heidelberg University Hospital, Heidelberg, Germany; 14 Division of Hematology and Oncology, Mayo Clinic, Phoenix, AZ USA; 15 Division of Hematology/Oncology, University of California San Francisco, San Francisco, CA USA; 16 Division of Hematology, Mayo Clinic, Rochester, MN USA

**Keywords:** Myeloma, Myeloma

## Abstract

Venetoclax is efficacious in relapsed/refractory t(11;14) multiple myeloma, thus warranting investigation in light-chain amyloidosis (AL). This retrospective cohort includes 43 patients with previously treated AL, from 14 centers in the US and Europe. Thirty-one patients harbored t(11;14), 11 did not, and one t(11;14) status was unknown. Patients received a venetoclax-containing regimen for at least one 21- or 28-day cycle; the median prior treatments was three. The hematologic response rate for all patients was 68%; 63% achieved VGPR/CR. t(11;14) patients had higher hematologic response (81% vs. 40%) and higher VGPR/CR rate (78% vs. 30%, odds ratio: 0.12, 95% CI 0.02–0.62) than non-t(11;14) patients. For the unsegregated cohort, median progression-free survival (PFS) was 31.0 months and median OS was not reached (NR). For t(11;14), median PFS was NR and for non-t(11;14) median PFS was 6.7 months (HR: 0.14, 95% CI 0.04–0.53). Multivariate analysis incorporating age, sex, prior lines of therapy, and disease stage suggested a risk reduction for progression or death in t(11;14) patients. Median OS was NR for either subgroup. The organ response rate was 38%; most responders harbored t(11;14). Grade 3 or higher adverse events occurred in 19% with 7% due to infections. These promising results require confirmation in a randomized clinical trial.

## Introduction

Systemic monoclonal immunoglobulin light-chain amyloidosis (AL) is an acquired form of amyloidosis, in which unstable light chains are produced by a neoplastic monoclonal plasma cell population. These light chains form oligomers and bind to cellular and extracellular matrix proteins causing direct toxicity and amyloid deposition in organs such as the heart and kidneys^1,2^. Studies are limited regarding the annual incidence of AL, but estimates range from 3 to 13 cases per million person-years^2–9^.

While there has been researching into targeting different steps of amyloid formation^10^, the backbone of treatment in patients with AL remains suppression of the neoplastic plasma cell clone. Treatment paradigms are borrowed from multiple myeloma (MM) and include combination chemotherapy regimens^11–16^ though to date no FDA-approved regimen for the treatment of AL exists. Unfortunately <20% of patients are transplant-eligible^17^ and patients receiving high-dose melphalan with autologous stem cell transplant (ASCT) face a hematologic relapse rate of over 30% with a time to relapse of 2–4 years^18^. Additionally, recent data suggest high hospitalization rates, progression to dialysis, and early mortality from ASCT in AL patients with renal dysfunction^19^. Daratumumab, a monoclonal antibody targeting CD38 on plasma cells, has shown promising efficacy in the relapsed/refractory (RR) setting as monotherapy and in combination therapy^20–23^. Daratumumab is also currently being studied in a randomized phase 3 trial in combination with CyBorD (cyclophosphamide, bortezomib, and dexamethasone) versus CyBorD alone in the upfront setting (NCT03201965). Unfortunately, patients with AL develop significant toxicities when treated with immunomodulatory medications (such as thalidomide and lenalidomide), limiting their use^24,25^. As such, options remain bleak for patients who have progressed on chemotherapy, ASCT, and antibody therapy-based approaches.

Venetoclax (ABT-199) is an oral selective B-cell lymphoma 2 (BCL-2) inhibitor FDA-approved for the treatment of chronic lymphocytic leukemia (CLL) and acute myelogenous leukemia (AML)^26–28^ with biological rationale for evaluation in plasma cell disorders. Preclinical data demonstrated that all human myeloma cell lines sensitive to venetoclax were restricted to the Cyclin D1 (CCND1) subgroup (80% harboring t(11;14)) and expressed elevated BCL-2:myeloid cell leukemia-1 (MCL-1) ratios^29^. High expression of MCL-1 leads to venetoclax resistance, however, this can be overcome with the addition of bortezomib, which is known to inhibit MCL-1^30–32^. Dexamethasone favorably alters sensitivity to venetoclax by increasing expression of both BCL-2 and BCL-2-like protein 11 (Bim) and shifting binding of Bim to BCL-2^33^. Together, this provided a clinical rationale to study venetoclax monotherapy and in combination with bortezomib and steroids in MM. In a phase I study, venetoclax monotherapy was found to have a favorable safety profile and the greatest efficacy was noted in patients with heavily treated t(11;14) MM where 40% of patients had a hematologic response with a 27% VGPR/CR rate^34^. The results from a phase 1b study examining the use of venetoclax and bortezomib in patients with RR MM showed a hematologic response rate of 67% in all patients and 94% in patients with high BCL-2 expression (with a VGPR/CR rate of 66%)^35^.

While this led to excitement over a potential targeted therapy in MM, preliminary results of the BELLINI trial (NCT02755597), which examined the use of bortezomib and dexamethasone + /− venetoclax in RR MM, showed an increased hazard ratio (HR) for all patients of 2.207 (95% CI: 1.042–3.945) for overall survival (OS) in the venetoclax arm^36^, despite a significantly higher progression-free survival (PFS) in patients receiving venetoclax. The alarming impact on OS led to an FDA-hold on the study of venetoclax–bortezomib combination therapy in MM. Interim results presented at the American Society of Hematology 2019 annual meeting demonstrated an improved HR for PFS of 0.095 (95% CI: 0.020–0.458) and a trend toward OS improvement with a HR of 0.649 (95% CI: 0.129–3.253) in t(11;14) patients^37^, renewing interest of venetoclax in t(11;14) RR MM.

Although present in only 17% of patients with MM, up to 60% of AL patients harbor t(11;14)^38–42^. Given the biological rationale, many clinicians have been using venetoclax-containing regimens in patients with RR AL, with anecdotal evidence of efficacy demonstrated in case reports and small case series^43–46^. Unlike MM, t(11;14) AL patients are traditionally less sensitive to bortezomib^47^; however, a recent case series showed a very good partial response or complete response (VGPR/CR) rate of 88% (seven out of eight patients) in t(11;14) AL patients treated with ventoclax-containing regimens, with all three patients who were treated with a venetoclax/bortezomib backbone achieving VGPR/CR^48^. While promising, larger datasets are needed to validate response and provide a strong foundation of evidence for a prospective clinical trial. Here, we report a multicenter, international, retrospective cohort study regarding treatment with venetoclax monotherapy and combination therapies for RR AL.

## Methods

### Study population and design

We conducted a retrospective cohort study involving 43 patients with RR AL that was approved by the Columbia University Institutional Review Board (primary Institutional Review Board). Data Use Agreements were carried out with participating institutions as applicable. Data were collected from 14 centers in the United States and Europe (see Supplementary Information).

**Fig. 1:**
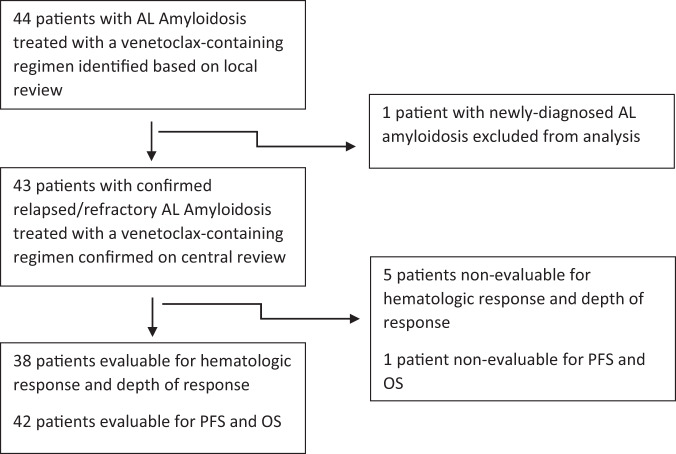
Study profile. Study profile for included patients.

Inclusion criteria consisted of patients with an established diagnosis of AL who had progressed on at least one prior line of therapy and were treated with a venetoclax-containing regimen for at least one 21- or 28-day cycle (Fig. 1).

Primary outcomes included OS, PFS, VGPR/CR rate, and toxicity (as assessed by Common Terminology Criteria for Adverse Events version 4.0) with pre-planned stratification by t(11;14) status. Other outcomes included hematologic response rate, organ response rate, and reason for discontinuation of therapy.

### Procedures

De-identified data extracted by local investigators and collected included patient demographics and clinical characteristics such as age, gender, light-chain subtype, t(11;14) status, the presence of high-risk cytogenetic features (del(17p) or monosomy 17, t(4;14), t(14;16), and gain (1q)), prior regimens, stage according to the Revised Prognostic Staging System for Light Chain Amyloidosis^49^, venetoclax-containing regimen along with information regarding the depth of hematologic response, organ response, toxicity, PFS, and OS following initiation of a venetoclax-based regimen. Patients were considered to harbor t(11;14) if it was detected on karyotyping or fluorescent in situ hybridization irrespective of the proportion of plasma cells harboring t(11;14).

Patient demographic and clinical characteristics information were then stratified based on t(11;14) status.

Treatment response was reported as determined by local investigators using the “New Criteria for Response to Treatment in Immunoglobulin Light Chain Amyloidosis Based on Free Light Chain Measurement”^50^. A pre-specified comparison of response and outcome between patients with and without t(11;14) was planned prior to data analysis. Organ responses were evaluated by criteria set forth by the Roundtable of Clinical Research in Immunoglobulin Light Chain Amyloidosis^51^.

### Statistical analysis

We created a 2 by 2 contingency table for VGPR/CR hematologic response outcomes by t(11;14)-positive and -negative subgroups. The odds ratio was reported, and then a Fisher’s Exact test was used to analyze the subgroups. For survival outcomes, PFS and OS curves were estimated using the Kaplan–Meier product limit estimator with medians and 95% confidence interval bounds. A data cutoff date was set for January 15, 2020, and patients were censored at their last follow-up date. PFS is defined as the time from the first day of treatment until hematologic progression or death. OS is defined as the time from the commencement of the venetoclax-containing regimen to death or last follow-up. A two-tailed log-rank test was used to perform survival comparisons between the patient groups. Hazard ratios (HRs) and 95% confidence intervals (CIs) between the patient groups were reported for survival outcomes using Cox regression methods. A multivariate analysis using Cox regression modeling was used to assess the effect of hematologic response and t(11;14) status on OS and PFS after adjusting for the effects of age, sex, stage, and prior lines of therapy. Chi-square tests and logistic regression were used to assess if t(11;14) status is significantly different between patient demographics and characteristics (Table 1). A *P* value of less than 0.05 was considered to indicate statistical significance. Data analyses were performed using R version 3.6.3 (R Foundation) and SAS 9.4 (SAS Institute).

**Table 1:** Patient demographics and characteristics.

	Entire cohort (*n* = 43)	t(11;14)	non-t(11;14)
*Sex*			
Male	28 (65%)	22 (71%)	5 (45%)
Female	15 (35%)	9 (29%)	6 (55%)
*Light-chain subtype*			
Lambda	29 (67%)	21 (68%)	7 (64%)
Kappa	14 (33%)	10 (32%)	4 (36%)
*Median age in years (range)*			
<65	19 (44%)	14 (45%)	4 (36%)
65–75	15 (35%)	11 (35%)	4 (36%)
>75	9 (21%)	6 (19%)	3 (27%)
Median prior lines of therpay (range)	3 (1–10)	3 (1–10)	5 (1–7)
Median stage prior to venetoclax (range)	2 (1–4)	2 (1–4)	2 (1–3)
*Prior therapies*			
Proteasome inhibitor (PI)	42 (98%)	31 (100%)	10 (91%)
Cyclophosphamide	34 (79%)	24 (77%)	9 (82%)
Daratumumab	25 (58%)	19 (61%)	6 (55%)
Lenalidomide	24 (56%)	17 (55%)	6 (55%)
Melphalan	13 (30%)	10 (32%)	3 (27%)
Pomalidomide	12 (28%)	5 (16%)	7 (64%)
Elotuzumab	3 (7%)	1 (3%)	2 (18%)
Bendamustine	2 (5%)	2 (6%)	0 (0%)
Liposomal doxorubicin	1 (2%)	0 (0%)	1 (9%)
Autologous stem cell transplant (ASCT)	9 (21%)	9 (29%)	0 (0%)

## Results

Forty-three patients with RR AL diagnosed between 2009 and 2019 at 14 institutions were included in the study. Patient demographics and disease characteristics with t(11;14) stratification are shown in Table 1. Patients were predominantly male (65%) with a median age of 66 years (range 49–83). Of all patients, 72% harbored t(11;14). Three patients harbored gain(1q), two of which concomitantly harbored t(11;14), and two patients harbored del(17p) or monosomy 17, one of which also harbored t(11;14).

Patients were heavily pretreated, having received a median of three (range 1–10) prior lines of therapy. Nearly, all patients received prior treatment with a proteasome inhibitor (98%) and the majority had prior treatment with cyclophosphamide (79%) and daratumumab (58%). In addition, 21% of patients had prior ASCT.

Overall patient demographics and disease characteristics were comparable between both t(11;14) and non-t(11;14) patients, and there were no statistically significant differences. Both subgroups were lambda light-chain subtype predominant- 68% in t(11;14) and 64% in non-t(11;14). Although t(11;14) patients had a median age younger than non-t(11;14) patients (66 vs. 72-year old), more t(11;14) patients were aged 75 or younger: 81% vs 73%. t(11;14) patients had a greater degree of male predominance (71% vs. 45%) and had fewer median prior lines of treatment (three vs. five). The median stage was two for both groups.

Venetoclax-containing regimens and dosage varied, however, the majority of patients (81%) were treated with either venetoclax + /− glucocorticoid (58%) or venetoclax + proteasome inhibitor (PI) + /− glucocorticoid (23%) therapy. Venetoclax dosing ranged from 100 mg daily to 800 mg daily.

**Fig. 2:**
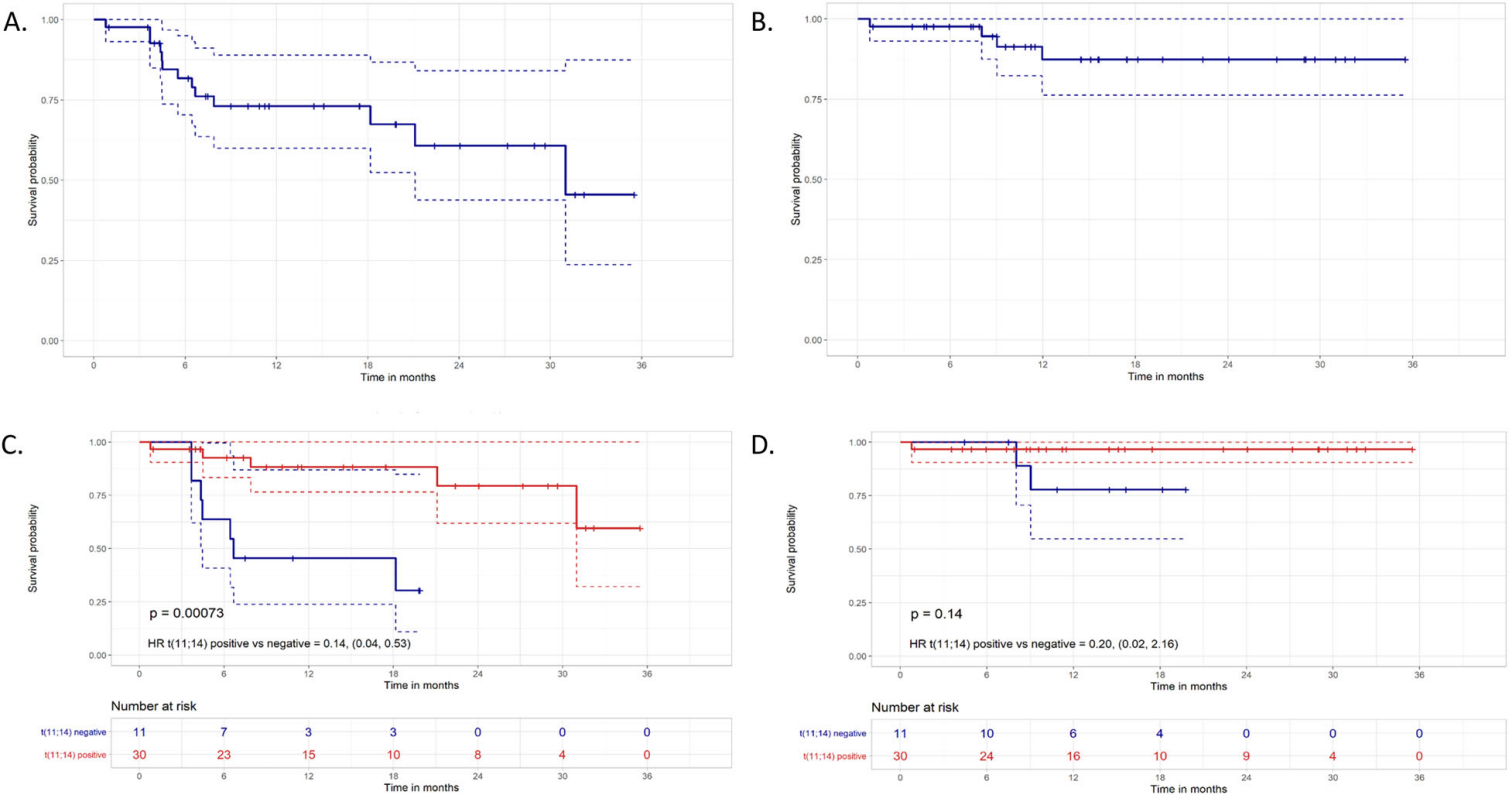
Kaplan–Meier curves for progression-free survival (PFS) and overall survival. **A** PFS for the unsegregated cohort. **B** OS for the unsegregated cohort. **C** PFS of t(11;14) patients vs non-t(11;14) patients. **D** OS of t(11;14) patients vs non-t(11;14) patients.

For the unsegregated cohort (*n* = 43), 42 patients were evaluable for PFS and OS (30 with t(11;14), 11 without t(11;14), one patient whose t(11;14) status is unknown and one with t(11;14) who was deemed non-evaluable for PFS and OS by local provider). The median follow-up was 14.5 months with a median PFS of 31.0 months (95% CI: 18.2 months—NR) and a median OS that was not reached (NR) (Fig. 2A, B). For the unsegregated cohort, 12-month PFS and OS were 78% and 93%, respectively. In the 30 evaluable patients harboring t(11;14), median PFS and OS were not reached and 12-month PFS and OS were 90% and 97%, respectively. For the 11 evaluable non-t(11;14) patients, median PFS was 6.7 months (95% CI: 3.7 months—NR), median OS was NR (Fig. 2C, D) and 12-month PFS and OS were 45% and 82% respectively. The HR for PFS was 0.14 (95% CI: 0.04–0.53), indicating an 86% reduction in risk for progression or death in patients harboring t(11;14). The HR for OS was 0.20 (95% CI: 0.02–2.16) favoring patients who harbored t(11;14), though not statistically significant.

**Fig. 3:**
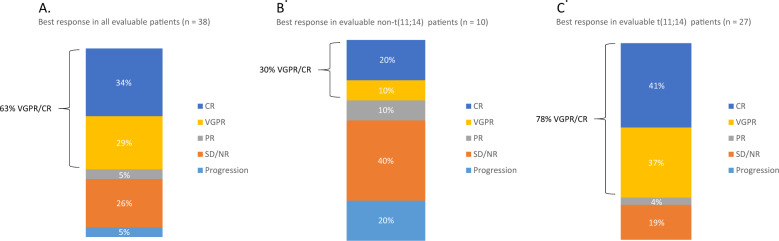
Hematologic response rate. **A** All evaluable patients, **B** response in non-t(11;14) patients, **C** response in t(11;14) patients.

Hematologic response rate (defined as partial response or better) was evaluable in 38 patients (27 t(11;14) patients, 10 patients without t(11;14), and one patient whose t(11;14) status was unknown) and was 68%, with a VGPR/CR rate of 63% and a median time to best response of 8.5 weeks. When stratifying by t(11;14) status, 81% (22 out of 27) of patients with t(11;14) achieved at least a PR and 78% (21 out of 27) a VGPR/CR. In ten evaluable non-t(11;14) patients, 40% (four patients) responded with at least a PR and 30% (three patients) with a VGPR/CR (Fig. 3A–C). Patients harboring t(11;14) were 88% more likely to attain a VGPR/CR compared to those without the translocation (odds ratio 0.12, 95% confidence interval: 0.02–0.62, *P* = 0.017). Of the 25 daratumumab-refractory patients, 12-month PFS and OS were 80% (84% in t(11;14) disease) and 92% (95% in t(11;14) disease), respectively. Twenty-two daratumumab-refractory patients were evaluable for hematologic response (16 t(11;14) patients and six non-t(11;14) patients) and 73% of patients had a hematologic response (75% in t(11;14) patients and 67% in non-t(11;14) patients) with a VGPR/CR rate of 64% (69% in t(11;14) patients and 50% in non-t(11;14) patients).

Thirty-five patients had full demographic and disease characteristics available and were evaluable for PFS, OS, and hematologic response. When stratified by t(11;14) status and adjusted for age, sex, prior lines of therapy, and disease stage, the data are highly suggestive that patients harboring t(11;14) have a reduction of risk for progression or death (HR 0.292, 95% CI: 0.046–1.855, *P* = 0.192) compared to non-t(11;14) patients.

Multivariate analysis revealed that hematologic response was significant for predicting for risk of progression or death (*P* = 0.013) for the follow-up period, and patients with a PR or VGPR/CR showed a reduction in risk of progression or death in comparison to non-responders (HR for PR: 0.031, 95% CI: 0.001–0.816; HR for VGPR/CR 0.011, 95% CI: 0.001, 0.179). Hematologic response at 12 months was also significant for predicting the risk of progression and death (*P* = 0.045); patients with a PR or VGPR/CR showed a reduction in risk of progression or death in comparison to non-responders (HR for PR: 0.049, 95% CI: 0.002–1.495, *P* = 0.084; HR for VGPR/CR 0.017, 95% CI: 0.001, 0.323). Patients achieving VGPR/CR also had an improvement in risk of progression and death at 12 months compared to non-responders (HR 0.017, 95% CI: 0.001, 0.323). There was no statistically significant difference in OS by t(11;14) status or hematologic response for either the follow-up period or at 12 months.

Data for cardiac, renal, neurologic, or hepatic organ involvement was available in 36 patients, 32 of which were evaluable for organ response. Of these 32 patients, 12 (38%) patients had responses in at least one listed organ system. Of the 12 patients with organ response, 10 harbored t(11;14) (83%) and two (17%) were non-t(11;14). Of 20 evaluable patients with cardiac involvement, six patients (30%) had organ response. Of 20 evaluable patients with renal involvement, eight patients (40%) had organ response. Of four evaluable patients with neurologic involvement, none had a response. Two patients had hepatic involvement however neither met the criteria for hepatic response. Other organs involved pulmonary (two), skin (two), lip (one), salivary glands (one), and soft tissue (one). One patient had acquired AL-related Factor X deficiency, which reversed following treatment with venetoclax and achievement of VGPR.

In the t(11;14) patients evaluable for hematologic response (*n* = 27), 15 (56%) received venetoclax + /− glucocorticoid therapy and experienced a VGPR/CR rate of 73% (7 CR, 4 VGPR). In this same subgroup, five patients (19%) were treated with venetoclax + PI + /− glucocorticoid and experienced a VGPR/CR rate of 100% (2 CR, 3 VGPR). In non-t(11;14) patients evaluable for hematologic response (*n* = 10), four (40%) were treated with venetoclax + PI + /− glucocorticoid with one patient having a hematologic response which was a VGPR (VGPR/CR rate of 25%). In this same population, five (50%) were treated with venetoclax + /− glucocorticoid with the hematologic response in three and two achieving VGPR/CR (40%).

Toxicity evaluation revealed that eight of 43 patients (19%) experienced a grade 3 or higher non-hematologic adverse event attributed to therapy (Table 2) and eight of 43 patients (19%) discontinued treatment due to toxicity. The most common non-hematologic adverse events were infection (35% of patients with 7% grade 3 or higher), diarrhea (26% of patients with 5% grade 3 or higher), and fatigue (19% of patients none of which were grade 3 or higher). The most common hematologic adverse event was thrombocytopenia (9% of patients with 5% grade 3 or higher) (Table 2). Though not neutropenic, one patient died from sepsis as a consequence of venetoclax therapy. In addition, one death due to heart failure in this cohort occurred within four weeks of starting venetoclax; however, this was not attributed to therapy.

**Table 2:** Toxicity.

	Entire cohort (*n* = 43)		t(11;14)		Non-t(11;14)	
	All grades	Grade 3 or higher	All grades	Grade 3 or higher	All grades	Grade 3 or higher
*Gastrointestinal*						
Diarrhea	11 (26%)	2 (5%)	8 (26%)	1 (3%)	3 (27%)	1 (9%)
Nausea	3 (7%)	0 (0%)	3 (10%)	0 (0%)	0 (0%)	0 (0%)
Abdominal pain	1 (2%)	1 (2%)	0 (0%)	0 (0%)	1 (9%)	1 (9%)
Unspecified	2 (5%)	0 (0%)	2 (6%)	0 (0%)	0 (0%)	0 (0%)
*Central nervous system*						
Fatigue	8 (19%)	0 (0%)	4 (13%)	0 (0%)	3 (27%)	0 (0%)
Dizziness	1 (2%)	0 (0%)	0 (0%)	0 (0%)	1 (9%)	0 (0%)
*Cardiovascular*						
Chest pain	1 (2%)	0 (0%)	1 (3%)	0 (0%)	0 (0%)	0 (0%)
Dyspnea	1 (2%)	0 (0%)	1 (3%)	0 (0%)	0 (0%)	0 (0%)
*Musculoskeletal*						
Shakiness	1 (2%)	0 (0%)	1 (3%)	0 (0%)	0 (0%)	0 (0%)
*Dermatological*						
Rash	2 (5%)	0 (0%)	1 (3%)	0 (0%)	1 (9%)	0 (0%)
*Hematological*						
Thrombocytopenia	4 (9%)	2 (5%)	3 (10%)	1 (3%)	1 (9%)	1 (9%)
Leukopenia	1 (2%)	0 (0%)	1 (3%)	0 (0%)	0 (0%)	0 (0%)
Neutropenia	1 (2%)	0 (0%)	0 (0%)	0 (0%)	1 (9%)	0 (0%)
*Infection*						
Upper respiratory infection	5 (12%)	0 (0%)	4 (13%)	0 (0%)	1 (9%)	0 (0%)
Sepsis	2 (5%)	2 (5%)	1 (3%)	1 (3%)	1 (9%)	1 (9%)
Pneumonia	1 (2%)	0 (0%)	1 (3%)	0 (0%)	0 (0%)	0 (0%)
Bronchitis	1 (2%)	0 (0%)	1 (3%)	0 (0%)	0 (0%)	0 (0%)
Influenza	1 (2%)	0 (0%)	0 (0%)	0 (0%)	1 (9%)	0 (0%)
Chest infection (unspecified)	1 (2%)	0 (0%)	0 (0%)	0 (0%)	0 (0%)	0 (0%)
Colitis	1 (2%)	1 (2%)	1 (3%)	1 (3%)	0 (0%)	0 (0%)
Cellulitis	1 (2%)	0 (0%)	0 (0%)	0 (0%)	1 (9%)	0 (0%)
Shingles	1 (2%)	0 (0%)	1 (3%)	0 (0%)	0 (0%)	0 (0%)
Styes	1 (2%)	0 (0%)	1 (3%)	0 (0%)	0 (0%)	0 (0%)

## Discussion

This multicenter, international, retrospective cohort study is the largest study, to our knowledge, that reports on outcomes of patients with RR AL treated with venetoclax-containing regimens. Given the retrospective nature and the heterogeneity of regimens and dosage used, the data reported have certain limitations; however, important insights can be gleaned. First, it is important to note that this is the largest cohort to report on targeted therapy for patients with AL based on cytogenetics. Stratification by t(11;14) demonstrates a large and statistically significant difference in high quality responses (VGPR/CR 78% vs. 30%) favoring t(11;14) patients with comparable hematologic responses in daratumumab-refractory patients. While OS was not reached for either subgroup due to a short follow-up period, the Kaplan–Meier survival curves trended toward a better response in t(11;14) patients. PFS analysis revealed a significant 86% reduction in risk of progression or death in t(11;14) patients (71% after multivariate adjustment).

While patient demographics and characteristics were generally comparable between t(11;14) patients and non-t(11;14) patients, it is important to highlight some potentially important differences. First, while t(11;14) patients had a younger median age than non-t(11;14) patients, a greater proportion of t(11;14) patients were actually ≤75 years old. Additionally, although both subgroups were heavily pretreated, t(11;14) patients had fewer median prior lines of treatment than non-t(11;14) patients (3 vs. 5), though this is not statistically significant. The disease stage was the same for both subgroups and thus did not confound subgroup analysis.

Our analysis showed there was no statistical difference in PFS or OS between older and younger patients, sex, or prior lines of therapy in our cohort. However, disease stage did show a statistically significant increase in the risk of progression or death with each increase in stage (HR 2.45, 95% CI: 1.06, 5.67, *P* = 0.036).

While there is conflicting data in the literature about the prognostic relevance of t(11;14) in AL^23,52^, our data suggest that the presence of this genetic aberration is a good biomarker for response to venetoclax. In plasma cell dyscrasias, t(11;14) is associated with a lower bone marrow plasma cell count, with the involved clone generally being the main clone in AL compared to a subclone in non-AL plasma cell dyscrasias^53^. In MM, t(11;14) patients are characterized by lower levels of monoclonal protein as well as more mature plasma cell morphology^54^. In addition, MGUS is associated with a higher incidence of t(11;14), suggesting that this translocation alone is not sufficient for plasma cells to acquire full malignant potential and secondary cytogenetic aberrations are required for myeloma progression^53,55,56^. It seems that overexpression of BCL-1 and BCL-2 associated with t(11;14) inhibits apoptosis and BCL-2 sequestration by venetoclax allows plasma cells to undergo apoptosis and more effectively induces deep and long-lasting remissions in “low malignancy” plasma cell dyscrasias like AL. Given the retrospective nature of this study, the results require confirmation by a prospective clinical trial, though given the high proportion of AL patients harboring t(11;14), venetoclax may have larger therapeutic implications than in MM.

Other important findings in this study are OS and PFS for patients with RR AL, regardless of t(11;14) status. Given the rarity of AL, and even more infrequent encounters with RR disease, data are lacking regarding PFS and OS in this patient population. Published data for patients with RR AL treated with daratumumab-based therapy show a 10-month PFS of 89%^21^ and a 10-month OS between 80% and 94%^21,57^. More than half of our patients were daratumumab-refractory and had a median of three prior lines of treatment. With a 12-month PFS and OS of 78% (90% in t(11;14) disease) and 93% (97% in t(11;14) disease) in the overall cohort respectively and a 12-month PFS and OS of 80% (84% in t(11;14) disease) and 92% (95% in t(11;14) disease) in daratumumab-refractory patients, we believe our results compare favorably.

While overall grade 3 or higher toxicity was noted to be 19% with a large portion being infection-related (7%), other grade 3 or higher toxicities were rare. The impact of administering prophylactic intravenous immunoglobulin and antibiotic regimens for patients in this cohort is not known, and this approach may be a potential way to abrogate infection risk, which could be examined in a randomized clinical trial. Toxicity did not appear to differ based on t(11;14) status. One patient died as a consequence of therapy (venetoclax + bortezomib + dexamethasone) due to sepsis and was not neutropenic at the time. The second death in the cohort was due to heart failure but was not deemed to be due to therapy per the primary investigator. Though early death cannot be fully assessed as patients in this study must have completed at least one cycle of venetoclax therapy, one death occurred within four weeks in this cohort.

We believe the findings of our study compare favorably to the toxicity profile seen in the BELLINI and NCT01794520 (a Phase I/II trial examining venetoclax + dexamethasone) trials for patients with RR MM. In BELLINI, patients treated with venetoclax + bortezomib + dexamethasone were found to have a rate of serious adverse events of 51% and a rate of serious infection of 30%, with 18% of patients developing neutropenia^37^, though the high rates of serious infection and increased HR for OS in the venetoclax arm was not seen in the phase 1b trial^35^. In the 31 patient Phase II NCT01794520, six patients (19%) died (five due to progressive disease, one from an adverse event) and three patients developed sepsis (10%). In addition, the most common grade 3 or 4 treatment-emergent adverse events were lymphopenia (32%), thrombocytopenia (11%) and hypertension (10%)^58^. While both patients in this data set and the discussed clinical trials were heavily pretreated, the favorable toxicity profile seen here may be due to better bone marrow reserve in AL patients who tend to have a more indolent neoplastic plasma cell clone than patients with MM. In addition, the toxicity profile of venetoclax-based combinations in this study compares favorably to well-established safety data in newly diagnosed CLL in combination with obinutuzumab^59^ and in combination with a hypomethylating agent in newly diagnosed elderly patients with AML^60^.

The most commonly used regimens were venetoclax + /− glucocorticoid and venetoclax + PI + /− glucocorticoid (see Supplementary Information). Both regimens were highly efficacious, especially in t(11;14) patients, and provided high rates of VGPR/CR. While venetoclax + /− glucocorticoid had a VGPR/CR rate of 73% (*n* = 15), venetoclax + PI + /− glucocorticoid had a VGPR/CR rate of 100% (*n* = 5), suggesting potential synergy between venetoclax and PIs in t(11;14) patients. The numbers, however, are small and thus a definitive conclusion cannot be drawn.

For patients evaluable for organ response, a high proportion (38%) achieved a response including 30% of patients with cardiac involvement and 40% of patients with renal involvement. It is important to note that some of the evaluable patients had improvement in organ function biomarkers but did not quite meet the criteria for partial organ response. In addition, some of these patients were still receiving treatment with venetoclax and continuing to have hematologic response and reduction in involved-organ biomarkers, so it is unclear at this time if they will eventually meet the criteria for organ response, which is typically delayed in AL. These data suggest that venetoclax can prevent and even improve organ dysfunction in patients with AL.

This study has several limitations aside from its retrospective nature. Given the heterogeneity of venetoclax-containing regimens, a wide range of dosages used, and irregular follow-up, identifying the optimal venetoclax combination with drug dosing was not possible. Additionally, the lack BCL-2 expression data make it unclear whether all t(11;14) AL patients overexpressed BCL-2 and if any of the non-t(11;14) patients who had good responses concurrently overexpressed BCL-2, perhaps by another mechanism. The lack of minimal residual disease data also limits the depth of response analysis and prognostic implications. Finally, the toxicities related to venetoclax-based regimens may have been underreported given the lack of a formal process to capture the adverse effects in a cohort that was approached retrospectively.

Taken together, however, the results of our multi-institutional study indicate a strong rationale toward a biomarker-driven approach for AL patients. Though our findings demonstrate the efficacy of venetoclax in the RR setting, they additionally serve as a platform for targeted treatment in newly diagnosed t(11;14) AL. Larger prospective studies are required to validate our findings that promote a new and convenient therapeutic approach, with a manageable toxicity profile and the potential to favorably alter the clinical course of AL.

## Data sharing statement

For original data please contact Vikram Premkumar (vikram.premkumar@gmail.com). Data including all individual participant data following de-identification will be available to researchers who provide a methodologically sound proposal, to achieve the aims of the approved proposal. The study protocol is available upon request. Data will be available beginning 9 months and ending 36 months following publication of the article.

## Supplementary information

Supplement

Reproducibility Checklist
