# Supplementary material for: Venetoclax induces deep hematologic remissions in t(11;14) relapsed/refractory AL amyloidosis
Source: Blood Cancer J. 2021 Jan 11;11(1):10. doi: 10.1038/s41408-020-00397-w (PMC7801694; doi:10.1038/s41408-020-00397-w)
Supplement: Supplementary file 1 — Supplement [file 41408_2020_397_MOESM1_ESM.docx]

**Supplemental Information**

**Methods**

**Study Population and Design**

Participating institutions included Columbia University Medical Center (New York, NY, USA), Mayo Clinic (Rochester, MN, USA and Phoenix, AZ, USA), University of California San Francisco (San Francisco, CA, USA), Mount Sinai Hospital (New York, NY, USA), Stanford University Medical Center (Palo Alto, CA, USA), University College Hospital (London, UK), Centre Hospitalier Universitaire à Limoges (Limoges, France), Hôpital Saint-Louis (Paris, France), Boston University Medical Center (Boston, MA, USA), Tufts Medical Center (Boston, MA, USA), City of Hope Comprehensive Cancer Center (Duarte, CA, USA), Cleveland Clinic (Cleveland, OH, USA) and Universitätsklinikum Heidelberg (Heidelberg, Germany).

**Results**

The venetoclax regimens used were: venetoclax +/- glucocorticoid (56%), venetoclax + PI +/- glucocorticoid (26%), venetoclax + Daratumumab + IMiD +/- glucorticoid (7%), venetoclax + PI + cyclophosphamide +/- glucocorticoid (5%), venetoclax + PI + IMiD + glucocorticoid (2%), venetoclax + Daratumumab (2%) and venetoclax + PI + Daratumumab (2%).
